# Ratio between Right Ventricular Longitudinal Strain and Pulmonary Arterial Systolic Pressure: Novel Prognostic Parameter in Patients Undergoing Cardiac Resynchronization Therapy

**DOI:** 10.3390/jcm10112442

**Published:** 2021-05-31

**Authors:** Silvia Deaconu, Alexandru Deaconu, Alina Scarlatescu, Ioana Petre, Sebastian Onciul, Aura Vijiiac, Diana Zamfir, Gabriela Marascu, Corneliu Iorgulescu, Andrei Dan Radu, Stefan Bogdan, Radu Vatasescu

**Affiliations:** 1 Faculty of Medicine, Carol Davila University of Medicine and Pharmacy, 050474 Bucharest, Romania; si.deaconu@gmail.com (S.D.); ioana.petre@umfcd.ro (I.P.); sebastian.onciul@gmail.com (S.O.); aura.vijiiac@drd.umfcd.ro (A.V.); gabriela.marascu@yahoo.com (G.M.); dan.radu@umfcd.ro (A.D.R.); bogdan.stefan@umfcd.ro (S.B.); radu.vatasescu@umfcd.ro (R.V.); 2 Cardiology Department, Clinical Emergency Hospital, 014461 Bucharest, Romania; alina.scarlatescu@gmail.com (A.S.); diana_zam74@yahoo.com (D.Z.); iorgulescu_corneliu@yahoo.com (C.I.)

**Keywords:** right ventricle strain, speckle tracking, pulmonary systolic arterial pressure, right ventricular function, cardiac resynchronization therapy, heart failure, right ventricular arterial coupling, pulmonary circulation

## Abstract

Background: We aimed to evaluate whether right ventricle (RV) longitudinal strain indexed to pulmonary arterial systolic pressure (PASP) has prognostic significance in patients undergoing cardiac resynchronization therapy (CRT). Methods: Patients undergoing CRT were prospectively included. The primary endpoint was adverse cardiovascular events (death and HF-related hospitalizations). RV global longitudinal strain (RVGLS) and RV free wall strain (RVfwS) were measured by speckle tracking and indexed to echocardiographic estimated PASP. Results: A total of 54 patients (64.0 ± 13.8 years; 58% male) were included. After 33 ± 12.9 months, the primary endpoint occurred in 18 patients. Baseline RVGLS/PASP and RVfwS/PASP showed good discriminative ability for response to CRT (AUC = 0.88, 95% CI (0.74–1) and AUC = 0.87, 95% CI (0.77–1)). RVGLS/PASP and RVfwS/PASP were significantly associated with high risk of events at univariate analysis (HR 0.039, 95% CI (0.001–0.8) *p* < 0.05, respectively HR = 0.049, 95% CI (0.0033–0.72), *p* < 0.05). Upon multivariate Cox regression analysis, RVGLS/PASP and RVfwS/PASP remained associated with high risk of events (HR 0.018, 95% CI (0.0005–0.64), *p* = 0.02 and HR 0.015, 95% CI (0.0004–0.524), *p* = 0.01) after correction for gender, etiology, QRS duration and morphology. **Conclusions:** Indexing RV longitudinal strain (global and free wall) by PASP provides a parameter, which independently identifies patients with high risk of cardiovascular events and predicts non-response to CRT.

## 1. Introduction

Cardiac resynchronization therapy (CRT) is an effective treatment for heart failure patients with reduced ejection fraction (HFrEF) [1]. The majority of CRT studies have focused on the evaluation of the left ventricle (LV). Nevertheless, several recent trials analyzed the role of the right ventricle (RV) in HFrEF patients, including those with left bundle branch block (LBBB) with indication of CRT. RV systolic dysfunction was shown to be an independent predictor of mortality in patients with HFrEF [2,3]. The presence of pulmonary hypertension (PH) also influences the response to CRT and survival rates [3,4]. Recent studies show that RV to pulmonary circulation coupling can be estimated non-invasively by echocardiography, using parameters of RV systolic function indexed to the pulmonary systolic pressure (PASP) [5,6]. TAPSE/PASP [5,7] and RV longitudinal strain/PASP [8] have been used to estimate RV to pulmonary circulation coupling and were proved to have prognostic value in HFrEF patients. Our study sought to analyze the role of indexing RV longitudinal strain (estimated by speckle tracking) to PASP in the setting of CRT. To our knowledge, this is the first attempt to establish the significance of the novel combined parameter RV strain/PASP ratio in patients undergoing CRT.

## 2. Methods

### 2.1. Study Patients

This is a prospective study which included 54 patients with HFrEF treated with CRT in the Cardiology Department of Clinical Emergency Hospital Bucharest, Romania in the period January 2017–November 2019. The study was approved by the Ethics Committee of the Clinical Emergency Hospital Bucharest (ethical approval No. 27031; date of approval 27 September 2018).

The inclusion criteria were patients with HFrEF, with indication for CRT according to current European Guidelines [1] and after complete coronary revascularization (in case of ischemic cardiomyopathy). The exclusion criteria consisted of a poor acoustic window, severe, free flowing tricuspid regurgitation, atrial fibrillation, severe pulmonary disease, pulmonary embolism, stenotic valvular disease, and patients who did not collaborate for follow-up. Documented clinical data were age, gender, NYHA class at baseline and follow up, etiology of LV dysfunction, co-morbidities. ECG data referred to the morphology of QRS (LBBB, and non LBBB), duration of PR, duration of QRS before and one year after CRT. All CRT implantations were done via the subclavian venous approach. Atrial leads were positioned in the right atrial appendage. RV leads were positioned in the RV mid-septal area. LV leads were introduced via the coronary sinus and targeted the lateral or postero-lateral cardiac vein.

Echocardiography was performed at baseline and repeated one year after CRT. The response to CRT was defined by echocardiography as more than a 15% reduction in LV end-systolic volume (LVESV) one year after CRT. Transthoracic echocardiography was performed by certified sonographers, using an ultrasound machine (Vivid E9, GE Vingmed Ultrasound AS, Horten, Norway) according to current international recommendations [9].

#### 2.1.1. Left Ventricle Echocardiographic Evaluation

LV volumes (end-diastolic and end-systolic) and LV ejection fraction (LVEF) were calculated using the biplane Simpson method. The LA end-systolic volume was calculated as an average using the area-length method in the four-chambers and two-chambers view. LV diastolic function parameters included the E/A ratio and ratio between early ventricular wave filing (E) and early myocardial velocity by Tissue Doppler Imaging (e), peak tricuspid velocity and left atrial indexed volume [10,11].

#### 2.1.2. Right Ventricle Echocardiographic Evaluation

TAPSE was measured in the apical four-chamber view using the M-mode through the lateral tricuspid annulus as the peak excursion of the tricuspid annulus from the end of diastole to end systole [12]. PASP was estimated using the tricuspid regurgitant velocity (TRV) and right atrial pressure (RAP) as follows: 4(TRV^2^) + RAP. RAP was estimated based on inferior vena cava (IVC) measurements: RAP= 5 mmHg (IVC diameter ≤ 21 mm, with >50% collapse), RAP =10 mmHg (IVC diameter ≤ 21 mm with <50% collapse or > 21 mm with >50% collapse), RAP =15 mmHg (IVC diameter > 21 mm, with <50% collapse) [12].

**Figure 1:**
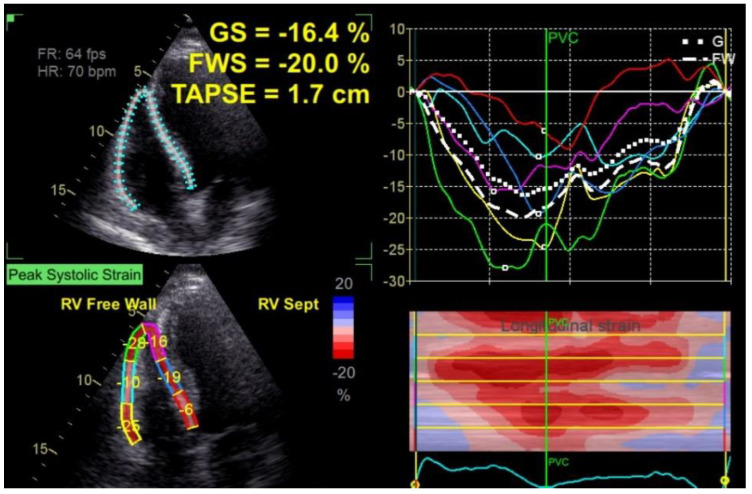
Calculation method of RV global longitudinal strain (GS) and free wall strain (FWS) using dedicated software.

The tricuspid S peak systolic velocity (S wave) was measured using Tissue Doppler Imaging at the tricuspid annulus, obtained from the apical view. The Tei index (the myocardial performance index for the RV) was calculated as the isovolumic time to the ejection time ratio. We used the pulsed wave Doppler method. The RV fractional area change (RV-FAC) was calculated using the following formula: RV end-diastolic area—RV end-systolic area)/RV end-diastolic area × 100. The RV area was traced in diastole and systole in the four-chamber view focused on the RV. The RV longitudinal strain was measured using two-dimensional images acquired from the four-chamber view for offline analysis using the strain software (EchoPAC, General Electric Vingmed Ultrasound). In order to determine the RV longitudinal strain, the endocardial border of the RV was traced manually and tracked by the software. RV global longitudinal strain (RVGLS) was calculated as the average of 6 segmental values of the lateral wall and interventricular septum and RV free wall strain (RVfwS) was calculated as the average of the 3 segments of the RV free wall. Both values were provided automatically by the software (Figure 1).

### 2.2. Follow-Up Period

Following CRT implantation, patients were tracked for adverse cardiovascular events. The documented events were cardiac death and hospitalizations due to HF. The total follow-up period was 33 ± 12.9 months after CRT.

### 2.3. Statistical Analysis

The results were expressed as mean ± standard deviation for continuous variables and percentage for categorical variables. The Pearson coefficient was used for establishing correlations between parameters before CRT. Receiver operator characteristic (ROC) curves were plotted for RV GLS/PASP and RV fwS/PASP, TAPSE/PASP, RVGLS, RVfwS to determine the area under the curve (AUC), and identify the optimal dichotomous threshold, specificity and sensitivity. Differences between echocardiographic parameters were analyzed with the paired sample t-test, independent t test. One way ANOVA was used to analyze differences of RVGLS/PASP and RVfwS/PASP among NYHA classes. A *p* value < 0.05 was considered significant. Univariate and multivariate Cox regression analyses were used to assess the prognostic value echocardiography parameters. The multivariate analysis model was built considering previous data showing that morbidity and mortality after CRT are influenced by etiology, gender, QRS width, and morphology [1]. The concordance index was calculated using test proportional hazard assumptions. The Kaplan–Meier analysis was used to assess the differences in survival among subjects according to dichotomous classification of the variables. All analyses and graphs were performed using STATA Statistical Software (StataCorp, College Station, TX, USA; version 16 for macOS).

## 3. Results

### 3.1. Study Data

The main characteristics of the overall cohort and according to the presence of adverse cardiovascular events (death and HF hospitalizations) are summarized in Table 1. Most of the patients were male (58%), NYHA class III and IV (73.68%), with typical LBBB on ECG. 28.8% of patients had ischemic etiology of HF.

**Figure 2:**
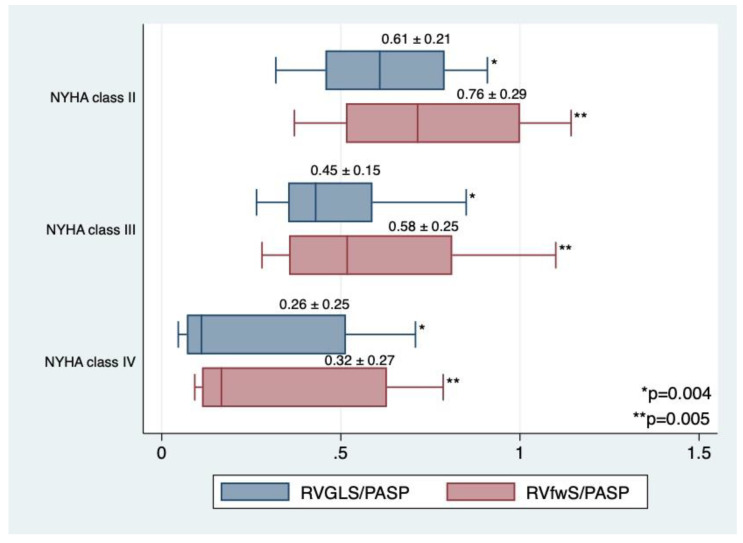
Mean values of RVGLS/PASP and RVfwS/PASP among NYHA classes at baseline.

At baseline, there are significant differences of RVGLS/PASP and RVfwS/PASP among NYHA classes, with the lowest values reported in the NYHA class IV (Figure 2).

Lower values of RVGLS/PASP are associated with high values of E/e ratio (r = −0.46, *p* = 0.02).

### 3.2. Evolution One Year after CRT

One year after CRT, 33 (61.2%) patients were volumetric responders. The evolution of different parameters after one year according to CRT response is summarized in Table 2.

**Figure 3:**
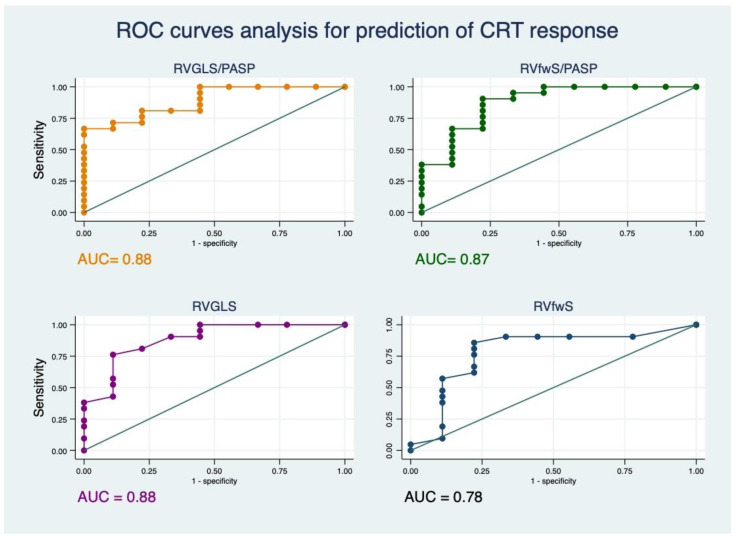
ROC curves analysis for prediction of CRT response for different variables. ROC: receiver operator characteristic, AUC: area under the curve, RVGLS: RV global longitudinal strain, PASP: pulmonary artery systolic pressure, RVfwS: RV free wall strain.

Using ROC curves analysis, we reported the predictive value for CRT response for different variables. RVGLS/PASP reported an AUC = 0.88, 95% CI [0.77–1] and RVfwS/PASP an AUC = 0.87, 95% CI [0.72–1], shown in Figure 3.

TAPSE/PASP reported an AUC of 0.83, 95% CI [0.61–1]), RVGLS an AUC of 0.88, 95% CI [0.74–1]) and RVfwS an AUC of 0.78, 95% [0.57–0.99]).

The best cut-offs for predicting response to CRT are as follows:

- RVGLS/PASP of 0.35 with sensibility of 80% and specificity of 77%.
- RVfwS/PASP of 0.41 with sensibility of 80% and specificity of 77%.
- RVGLS of −11% with sensibility of 80% and specificity of 77%.
- RVfwS of −13% with sensibility of 80% and specificity of 77%.

Higher values of RVGLS/PASP and RVfwS/PASP were associated with a higher probability for CRT response after one year at univariate logistic regression analysis, reporting an OR of 17.3 for RVGLS/PASP (95% CI 1.5–20, *p* < 0.001) and 12.5 for RVfwS/|PASP (95% CI 1.6–15, *p* < 0.001). RVGLS and RVfwS alone reported an OR of 1.47 for response to CRT (95% CI 1.1–1.98, *p* = 0.003) and respectively OR of 1.17 (95% CI 1.01–1.36, *p* = 0.01).

Unlike CRT non-responders, CRT responders exhibited reverse remodeling of LA volume in addition to LV remodeling. In the whole cohort, there was an increase in RV GLS at one year (from −12 ± 5.08% to −14 ± 5.15%, *p* = 0.008), especially in CRT responders. RV fwS did not improve significantly at one year and neither did RVGLS/PASP and RVfwS/PASP.

### 3.3. Survival Analysis

A total of 18 patients had events (14 hospitalizations and 4 deaths) at follow up.

Using univariate Cox analysis, both RVGLS/PASP and RVfwS/PASP were significantly associated with high risk of adverse events (HR 0.039, 95% CI [0.001–0.8] *p* < 0.05, respectively HR = 0.049, 95% CI [0.0033–0.72], *p* < 0.05, Table 3).

Upon multivariate Cox regression analysis, RVGLS/PASP and RVfwS/PASP remained associated with high risk of events (HR 0.018, 95% CI [0.0005–0.64], *p* = 0.02 and respectively HR 0.015, 95% CI [0.0004–0.524], *p* = 0.01) after correction for gender, etiology (ischemic), presence of typical LBBB, and duration of QRS.

TAPSE/PASP remained significantly associated with a high risk of events using Cox univariate analysis (HR: 0.046; 95% CI [0.004–0.45], *p*= 0.009) but at the limit of significance in the clinical multivariate model (HR: 0.045; 95% CI [0.001–1.02], *p*= 0.053).

**Figure 4:**
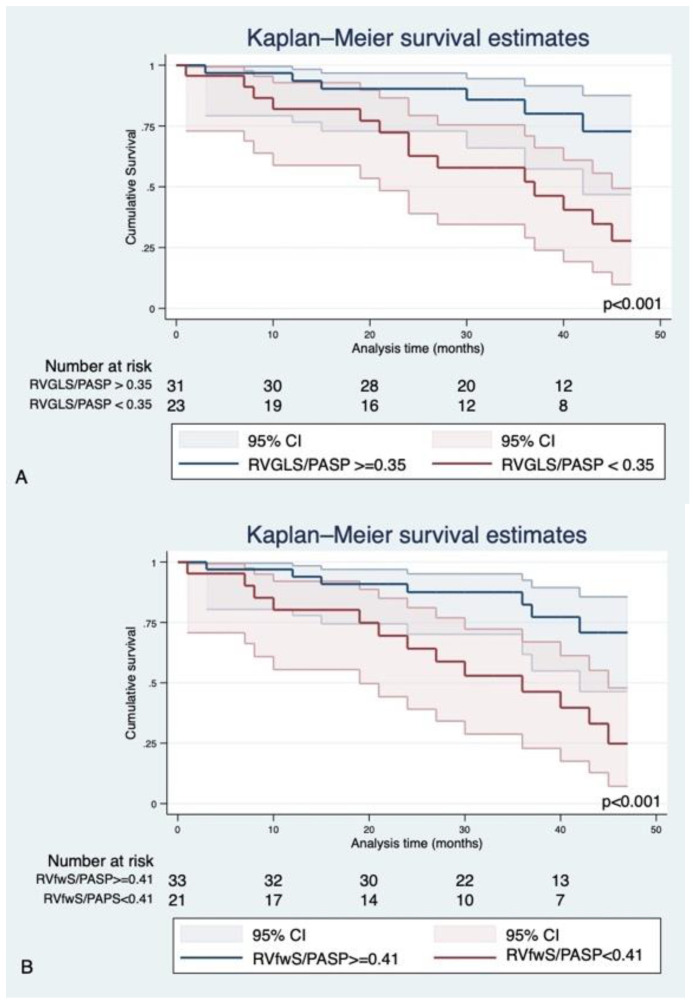
Kaplan–Meier survival analysis according to cut-offs identified for prediction of CRT response for variables RVGLS/PASP (**A**) and RVfwS/PASP (**B**).

Kaplan–Meier curves clearly illustrate the difference in cardiovascular events among patient groups according to the cut-offs identified to predict CRT response (0.35 for RVGLS/PASP and 0.41 for RVfwS/PASP, Figure 4A,B).

## 4. Discussion

Our study shows that evaluation of RV longitudinal strain (global and free wall) in the setting of CRT and further indexing it to PASP offers valuable prognostic information. Our main conclusions are the following:

- Low values of RVGLS/PASP and RVfwS/PASP are independently associated with a high risk of cardiovascular events (when corrected for gender, ischemic etiology, QRS morphology and duration).
- Low values of RVGLS/PASP and RVfwS/PASP predict non-response to CRT.

The concept of RV to pulmonary circulation coupling refers to the relationship between RV contractility and RV afterload. In the setting of a failing LV, the rising of pulmonary venous pressure leads to an initial augmentation of RV contractility and remodeling as a reaction to the increased afterload [13]. Later, however, when loading conditions become excessive, RV adaptation fails [13] and the uncoupling of RV from pulmonary circulation occurs. This process may reflect an advanced phase of HF evolution and this might be the explanation for its prognostic significance.

The RV to pulmonary circulation coupling may be evaluated by invasive catheterization using the pressure-volume loop-derived end-systolic/arterial elastance ratio [Ees/Ea] [6]. End-systolic elastance reflects RV contractility and when it becomes less than arterial elastance, which stands for the RV afterload, the Ees/Ea ratio decreases, leading to ventricular–arterial uncoupling, which is a physiological sign of RV failure [13].

Novel studies show that echocardiography can accurately estimate RV to pulmonary circulation coupling by combining parameters of RV systolic function indexed to pulmonary systolic arterial pressure [6,7]. RV systolic function can be evaluated by echocardiography, using multiple parameters due to the complex shape and contractility pattern [9]. This is why cardiac magnetic resonance remains the leading technique for evaluation of RV volumes and ejection fraction [14]. Among the echocardiographic parameters of RV systolic function, the most frequently used is TAPSE, which estimates the longitudinal systolic function of RV with the assumption that the regional basal segments and their systolic excursion toward the RV apex are representative for the overall RV function [12]. TAPSE correlates well with other methods of investigating the RV function [12].

Consequently, TAPSE indexed to PASP was the first choice in the attempt to evaluate RV to pulmonary circulation coupling by echocardiography. A study by Tello et al., performed by combining invasive data with echocardiography, showed that TAPSE/PASP emerged as an independent predictor of RV to pulmonary circulation coupling [6]. The same data were confirmed in patients with heart failure by Guazzi et al., who reported that the TAPSE/PASP ratio was taken as a noninvasive index of RV to pulmonary circulation coupling based on the correlation with invasively evaluated RV systolic elastance/arterial elastance (r = 0.35; *p* < 0.0001) [5]. Moreover, TAPSE/PASP emerged as a prognostic parameter in HF patients with a reduced and preserved ejection fraction; lower values of this ratio were associated with worse outcome [5,7]. In our cohort, TAPSE/PASP was significantly lower in patients who presented adverse cardiovascular events at follow up. A study by Braganca et al., performed in HFrEF patients and CRT, found that TAPSE/PASP predicts response to CRT [15].

The RV systolic function, however, can be evaluated using other parameters: tricuspid S wave velocity using Tissue Doppler Imaging, the RV fractional area change (RV FAC), the RV myocardial performance index, or the TEI index, which is a measurement of both systolic and diastolic RV function [12]. Kawata et al., for example, found that RV FAC may provide better prognostic information than TAPSE or the S wave in advanced heart failure patients with dilated cardiomyopathy [16]. In our study, RV FAC and S wave were significantly lower in CRT patients who developed adverse cardiovascular events. The Tei index did not exhibit a similar behavior. More recent echocardiographic parameters for RV systolic function consist of RV longitudinal strain evaluation by speckle tracking echocardiography and three dimensional (3D) echocardiography [12].

The novel method of speckle tracking imaging allows a more global quantification of RV deformation during the cardiac cycle [12]. The RV global longitudinal strain might be a promising non-invasive parameter to quantify RV function [8]. Therefore, Iacoviello et al. performed a study, which included 315 patients with HFrEF and estimated RV to pulmonary circulation coupling by indexing RV strain to PASP. Their conclusion was that RVSL (global and free wall) indexed to PASP provides a parameter of ventricular arterial coupling that is independently associated with an increased risk of mortality in HFrEF patients [8]. The best cut-offs for predicting 12-month mortality were 0.36 for RVGLS/PASP (sensitivity = 89%, specificity = 67%) and 0.65 for RVfwS (sensitivity = 89%, specificity = 54%).

In our study, lower values of RVGLS/PASP and RVfwS/PASP correlated with higher risk of events in univariate, but more importantly in multivariate, analysis when corrected for gender, etiology (ischemic), presence of typical LBBB, and duration of QRS. We chose this model upon multivariate analysis according to previous data showing that reverse remodeling after CRT is crucial for improvement in morbidity and mortality and it is influenced by etiology, gender, QRS width, and morphology [1]. The CRT response is more likely to occur in non-ischemic patients, female gender, with large QRS and LBBB morphology [1]. RVGLS and PASP alone are also predictors of cardiovascular events in this model of multivariate analysis, but the best concordance index is obtained when indexing RV strain to PASP.

TAPSE/PASP also correlated with a high risk of events but significantly only in univariate analyses. Before CRT, there were significant differences of RVGLS/PASP and RVfwS/PASP among NYHA classes with lowest values in NYHA class IV. We analyzed the role of this new parameters for predicting response to CRT and the AUC retained in ROC curves analysis was slightly better than for TAPSE/PASP. The cut off values obtained in our study for predicting CRT response were 0.35 for RVGLS/PASP (sensibility of 80% and specificity of 77%) and 0.41 for RVfwS/PASP (sensibility of 80% and specificity of 77%). The higher values of RVGLS/PASP and RVfwS/PASP were associated with CRT response using univariate logistic regression analysis.

The role of analyzing RV strain in CRT patients may have more potential, as very recent data show that in patients with LV systolic dysfunction and LBBB, there is abnormal contraction of the interventricular septum (IVS) with marked early systolic shortening and leftward motion that also affect RV contraction [17]. Storsten et al. investigated how LBBB and CRT modify RV free wall motion. The work gathers animal experimental data with human measurements in patients with non-ischemic cardiomyopathy, LBBB and a normal RV function [17] The study revealed an abnormal early systolic premature shortening of the RV free wall visible with the echocardiographic longitudinal strain analysis. Interestingly, in this study, CRT reduced or abolished this abnormal RV shortening, therefore increasing the RV workload [17]. Lumens et al. analyzed the mechanics of a failing heart with LBBB and treatment with CRT, using computer simulations with models of artificial hearts [18]. They confirmed the early systolic RV shortening in patients with LBBB and the fact that it is amenable by CRT. However, CRT did not lead to this mechanical change in the heart with additional RV failure [18].

In our study, on real-life data, the RVfwS did not change after CRT; however, RVGLS improved one year after CRT (*p* = 0.008), mostly in patients with LV reverse remodeling. Our study analyzed the evolution of RV function one year after CRT; therefore, the effect may be different from the acute changes induced by LBBB and CRT presented in the studies by Storsten and Lumens. None of the RV to pulmonary circulation coupling parameters (RVGLS/PAPS. RVfwS/PASP, TAPSE/PASP) were modified by CRT at one year follow-up. This may be explained mostly by the lack of significant decrease in PASP one year after CRT (Table 2). The contradictory results of the effect of CRT upon RV function [19,20] may be due to the fact that the RV workload seems to be increased by CRT [17], and therefore the RV evolution in time may differ among CRT patients depending on the RV function at baseline.

Our study has several limitations: it is a single center study including a small number of patients and, consequently, has a low event rate, which may affect the survival analysis. Another drawback is that we included only patients with good echocardiographic acoustic windows, which allowed the evaluation of all RV systolic function parameters TAPSE, tricuspid S wave, TEI index, RV FAC, RV strain and PASP, which can make our dataset more vulnerable to selection bias. Moreover, the calculation of PASP is dependent on the presence of tricuspid regurgitation and the estimation of right atrial pressure. In addition to the clinical and echocardiographic data, biologic markers (BNP, NT-proBNP) supporting improvement of HF would have added utility. However, our work provides preliminary data regarding a new echocardiographic parameter, which may provide clinical relevance, as it helps to select patients at high risk of adverse events who may not benefit from CRT.

## 5. Conclusions

Our study shows that, in the setting of CRT, indexing RV longitudinal strain (global and free wall) by PASP provides a novel parameter reflecting ventriculo-arterial coupling, which independently identifies patients with high risk of cardiovascular events and has good predictive value for CRT response.

## Figures and Tables

**Table 1:** Characteristics of the entire cohort and divided in two subgroups according to the occurrence of adverse cardiovascular events (death and HF hospitalizations).

Variables	Overall (*n* = 54)	Events (*n* = 18)	Without Events (*n* = 36)	*p* Value
Age (years)	64.0 ± 13.8	59.5 ± 13	66 ± 12.19	0.09
Male gender (%)	58	64.2	53.9	0.5
LBBB (%)	70.7	66.6	70.0	0.8
Ischemic etiology (%)	28.8	50	19.2	0.04
NYHA class (%)				0.03
II	26.32	15	32	
III	39.47	23.08	48	
IV	34.21	61.65	20	
QRS (ms)	170.27 ± 21.4	176 ± 31.07	169 ± 17.7	0.44
LV EDD (mm)	65 ± 10.1	72 ± 12	61 ± 6.6	0.009
LVEF (%)	28.4 ± 1.3	26.69 ± 18.8	29.5 ± 6.18	0.2
LVEDV (mL)	212.7 ± 17	251 ± 150.37	183 ± 75.7	0.04
LVESV (mL)	156 ± 94	198 ± 139.74	131 ± 64.69	0.03
E/e ratio baseline	14 ± 11.4	21.028 ± 17.9	10.67 ± 4.54	0.02
Tei index	0.57 ± 0.22	0.66 ± 0.22	0.54 ± 0.21	0.18
S wave (cm/sec)	0.11 ± 0.029	0.09 ± 0.028	0.11 ± 0.029	0.04
TAPSE/PASP (mm/mmHg)	0.7 ± 0.2	0.5 ± 0.27	0.80± 0.22	<0.001
TAPSE (mm)	21 ± 3.8	18.6 ± 3.62	22.3 ± 3.37	<0.001
PASP (mmHg)	34 ± 13.6	44.42 ± 17.14	29.73 ± 9.8	0.001
FAC RV (%)	41.5 ± 18.3	31 ± 20.5	47.83 ± 15.47	0.01
RVGLS (%)	12 ± 5.2	9.3 ± 5.67	13.9 ± 4.5	0.009
RVGLS/PASP	0.43 ± 0.23	0.27 ± 0.25	0.50 ± 0.21	0.005
RVfwS (%)	16 ± 7.45	11.8 ± 5.49	18 ± 7.54	0.01
RVfwS/PASP	0.54 ± 0.29	0.32 ± 0.21	0.65 ± 0.29	0.003

Events: Cardiac death and HF hospitalizations; LBBB: Left bundle branch block, NYHA: New York Heart Association; QRS: Duration of the QRS complex in the electrocardiogram, LVEF: Left ventricular ejection fraction, LVEDV: left ventricle end diastolic volume, E/e: ratio between early ventricular wave filing (E) and early myocardial velocity (e), PASP: Pulmonary artery systolic pressure, TAPSE: Tricuspid annular plane systolic excursion, FAC RV: Fractional area change right ventricle, Tei index: RV myocardial performance index, S wave: Systolic velocity al tricuspid annulus using Tissue Doppler Imaging. RVGLS: RV global longitudinal strain, PASP: Pulmonary artery systolic pressure. RVfwS: RV free wall strain.

**Table 2:** The evolution of different variables one year after CRT, according to CRT response.

	CRT Responders (*n* = 33)			CRT Non-Responders (*n* = 21)		
Variable	Baseline	One Year	*p* Value	Baseline	One Year	*p* Value
QRS (ms)	173 ± 16.8	136 ± 12.7	<0.001	177 ± 34.9	138.57 ± 16.7	0.005
LV EF (%)	30.06 ± 6.47	43.33 ± 7.90	<0.001	22.2 ± 6.98	21.9 ± 7.88	0.75
LAESV (mL)	72.43 ± 29.2	61.18 ± 20.2	0.02	127 ± 45.6	110 ± 41.7	0.9
RAESV (mL)	39 ± 13.17	41.33 ± 12.11	0.46	74.62 ± 30	72 ± 42.3	0.5
TAPSE (mm)	22.5 ± 3.03	24 ± 2.9	0.057	18.02 ± 3.9	19.2 ± 2.7	0.14
PASP (mmHg)	28 ± 5.57	29.63 ± 5.12	0.3	46 ± 20.2	41 ± 14.7	0.27
TAPSE/PASP (mm/mmHg)	0.8 ± 0.22	0.8 ± 0.17	0.5	0.50 ± 0.33	0.54 ± 0.26	0.7
RVGLS (%)	14.4 ± 3.7	16.3 ± 3.08	0.03	7.4 ± 4.5	8.6 ± 5.07	0.053
RVGLS/PASP	0.53 ± 0.19	0.56 ± 0.15	0.36	0.21 ± 0.17	0.28 ± 0.10	0.06
RVfwS (%)	17.2 ± 6.01	18.04 ± 5.14	0.52	10.44 ± 6.26	10.84 ± 6.18	0.69
RVfwS/PASP	0.61 ± 0.23	0.63 ± 0.23	0.15	0.28 ± 0.22	0.32 ± 0.24	0.15

QRS: Duration of the QRS complex in the electrocardiogram, LVEF: left ventricular ejection fraction, LAESV: left atrium end systolic volume, RAESV: right atrium end systolic volume, PASP: pulmonary artery systolic pressure, TAPSE: tricuspid annular plane systolic excursion, RVGLS: RV global longitudinal strain, PASP: pulmonary artery systolic pressure, RVfwS: RV free wall strain.

**Table 3:** Univariate and multivariate Cox regression survival analyses.

Variables	HR	C-Index	*p* Value	95% CI
**Univarate analysis**				
Gender (female)	0.69	0.50	0.55	0.20–2.32
Etiology (ischemic)	2.60	0.69	0.09	0.83–8.11
NYHA class	2.57	0.67	0.04	1.02–6.48
QRS duration	1.02	0.59	0.12	0.99–1.04
LBBB	1.10	0.49	0.88	0.28–4.21
TAPSE	0.82	0.69	0.02	0.69–0.96
RVGLS	0.88	0.61	0.06	0.78–1.00
RVfwS	0.91	0.63	0.09	0.82–1.01
PASP	1.03	0.78	0.02	1–1.07
TAPSE/PASP	0.046	0.73	0.009	0.004–0.45
RVGLS/PASP	0.039	0.65	0.03	0.001–0.8
RVfwS/PASP	0.049	0.70	0.02	0.003–0.72
**Multivariate analysis**				
Gender (female)	1.48		0.6	0.28–7.79
Etiology	4.63		0.05	0.94–22.7
QRS duration	1.01		0.18	0.99–1.03
LBBB	2.2		0.35	0.39–13.40
TAPSE/PASP	0.045	0.76	0.053	0.001–1.02
Gender (female)	2.82		0.2	0.40–19.86
Etiology	15.6		0.01	1.62–149
QRS duration	1.01		0.36	0.98–1.03
LBBB	4.93		0.13	0.61–39.39
RVGLS	0.83	0.78	0.04	0.70–0.99
Gender (female)	2.20		0.44	0.28–17.19
Etiology	15.32		0.02	1.33–175
QRS duration	1.01		0.21	0.99–1.03
LBBB	4.44		0.16	0.55–35.5
RVfwS	0.86	0.72	0.059	0.74–1.00
Gender (female)	1.35		0.7	0.24–7.48
Etiology	6.29		0.03	1.12–35.52
QRS duration	1.01		0.23	0.99–1.03
LBBB	3.24		0.23	0.45–23.13
PASP	1.05	0.75	0.03	1.00–1.10
Gender (female)	2.62		0.32	0.39–17.62
Etiology	15.3		0.01	1.85–127.56
QRS duration	1.01		0.20	0.99–1.03
LBBB	4.44		0.11	0.68–28.9
RVGLS/PASP	0.018	0.80	0.02	0.0005–0.64
Gender (female)	2.37		0.39	0.31–17.68
Etiology	20.5		0.01	1.98–212.62
QRS duration	1.01		0.99	0.99–1.04
LBBB	6.19		0.07	0.80–47.33
RVfwS/PASP	0.015	0.82	0.01	0.0004–0.52

NYHA: New York Heart Association, QRS: Duration of the QRS complex in the electrocardiogram, LBBB: typical left bundle branch block, TAPSE: tricuspid annular plane systolic excursion RVGLS: RV global longitudinal strain, PASP: pulmonary artery systolic pressure, RVfwS: RV free wall strain HR: hazard ratio, C-index: concordance index. CI: confidence interval.

## Data Availability

The data presented in this study are available on reasonable request from the corresponding author.
